# Translation of clinical practice to research: the VETS and ETHOS epidemiologic prospective cohorts

**DOI:** 10.3389/fcvm.2025.1577931

**Published:** 2025-07-10

**Authors:** Jonathan Myers, Peter Kokkinos, Immanuel Babu Henry Samuel, Charles Faselis, Ross Fletcher, Victor Froelicher

**Affiliations:** ^1^ Cardiology Division, Veterans Affairs Palo Alto Health Care System, Palo Alto, CA, United States; ^2^ Cardiology Division, Stanford University, Stanford, CA, United States; ^3^ Department of Cardiology, Veterans Affairs Medical Center, Washington, DC, United States; ^4^ Department of Kinesiology and Health, Rutgers University, New Brunswick, NJ, United States; ^5^ Veterans Affairs Medical Center, War Related Illness Study Center, Washington, DC, United States; ^6^ Henry M. Jackson Foundation for the Advancement Military Medicine, Bethesda, MD, United States; ^7^ Biomedical Research Artificial Intelligence and Neuroimaging Lab, Veterans Affairs Medical Center, Washington, DC, United States; ^8^ George Washington University School of Medicine and Health Sciences, Washington, DC, United States

**Keywords:** exercise testing, exercise testing (CPET), cardiovascular disease, cardiorespiratory fitness, epidemiology

## Abstract

For >30 years, the Exercise Testing and Health Outcomes Study (ETHOS) and the Veterans Exercise Testing Study (VETS) cohorts have contributed significantly to the understanding of the association between cardiorespiratory fitness (CRF), health outcomes, and the prevention of chronic disease. Multiple reports from these studies have consistently shown an inverse and graded association between higher CRF and the incidence of chronic conditions including cardiovascular disease, site-specific cancers, chronic kidney disease, rhythm disturbances, and neurological conditions. In addition, higher CRF is inversely related to health care costs. Among individuals whose CRF level improves over periods of time ranging from 5 to 7 years, improvements in health outcomes have been observed, and the converse is true among those who decrease CRF over time. The Veterans Administration Health Care System (VAHCS) has pioneered electronic medical records that have facilitated epidemiologic research and have provided the foundation for the ETHOS and VETS cohorts. The VAHCS is particularly suited for epidemiologic studies because patients can be accurately traced through VAHCS benefits services. These studies have helped formulate guidelines on exercise testing as well as recommendations from national and international health organizations on physical activity. In addition, they have provided strong support for efforts to reduce sedentary behavior, promote physical activity, and enhance CRF by public health organizations and healthcare systems in order to reduce the risk of chronic disease. This paper outlines the development of the ETHOS and VETS cohorts and highlights key studies contributing to our understanding of CRF as a critical health determinant.

## Introduction

Although there have been major advances in technologies related to the diagnosis of cardiovascular disease (CVD) in recent decades, the standard exercise test (ET) remains an important tool in the management of CVD because of its uniquely high yield of diagnostic and prognostic information. In fact, treatment guidelines continue to suggest that the exercise test should remain the first-choice modality when considering the presence of coronary artery disease (CAD) (1, 2). In particular, much has been published in recent years documenting the prognostic applications of the ET. A particularly powerful prognostic marker is exercise capacity. A growing number of studies have demonstrated that cardiorespiratory fitness (CRF) outperforms traditional risk factors such as smoking, hypertension, hyperlipidemia, obesity and diabetes in terms of predicting risk for adverse outcomes (3–5). The American Heart Association recently commissioned a Scientific Statement entitled, “Importance of assessing cardiorespiratory fitness in clinical practice: A case for fitness as a clinical vital sign” in which it states, “At a minimum, all adults should have CRF measured or estimated each year” (3). Subsequent expert commentaries and reviews of this topic have reinforced the concept that including CRF as part of routine clinical encounters should become a requisite component of disease prevention (4–11). These studies have focused not only on the relationship between CRF, all-cause mortality and incidence of CVD, but also outcomes related to chronic conditions including cancer, kidney disease, rhythm disturbances, COVID-19, neurological conditions, and cognitive decline. This has led multiple public health organizations to promote regular physical activity in order to improve CRF (12–16).

The support for the role of CRF in assessing health outcomes has required large populations along with detailed and accurate electronic medical records (EMRs). Such records were generally not available from large health care systems until the mid-1990s. The Veterans Affairs Health Care System (VAHCS) is considered a pioneer in adopting EMRs; it was one of the first health systems to implement EMR on a large scale, and the VAHCS had an influential role in transforming healthcare delivery through digital technology (17, 18). The VA Health Information Systems and Technology Architecture (VistA) was an early and highly influential example of applying EMRs in the US. Its success played a significant role in demonstrating the potential of EMRs to improve care coordination, reduce errors, enhance efficiency in healthcare, and facilitate epidemiologic research (19). In terms of applications to epidemiologic studies, the VAHCS EMR has been demonstrated to be more accurate than other health systems or publicly available data sets such as the Social Security Death Index (20, 21).

The VAHCS is also unique in that it ensures equal access to medical care independent of a patient's financial status. This has permitted more precise and complete assessments of the associations between CRF, mortality risk and other health outcomes as well as minimizing the influence of medical care disparities. In addition, the above-mentioned VAHCS EMR system facilitates risk-adjustment models, thereby increasing the accuracy in determining health outcomes. The VA is particularly suited for epidemiologic studies because patients usually stay within their catchment area and can easily be traced through VA benefits services. These features laid the foundation for the Exercise Testing and Health Outcomes (ETHOS) and Veterans Exercise Testing Study (VETS) cohorts. For >30 years, these epidemiologic studies, currently including >831,000 participants, have provided the opportunity to address numerous clinically relevant questions that apply to Veterans and the public. These large, prospective epidemiologic studies from the VAHCS that include detailed health information along with objective measures of CRF have been particularly important in advancing the understanding of the impact of CRF on the prevention of chronic disease. In the following, an outline of the development and contributions of the ETHOS and VETS cohorts to the assessment of health outcomes is presented.

## Methods

The VETS and ETHOS cohorts are prospective evaluations of Veteran subjects referred for exercise testing for clinical reasons, designed to address exercise test, clinical, and lifestyle factors and their association with health outcomes. Data collection is ongoing and evolving such that new and additional clinically relevant questions can be addressed.

### The VETS cohort

Beginning at the Long Beach VAHCS in the mid-1980s, a standard form for clinical and exercise test data gathering was implemented. It was first computerized using a bubble mark scoring test sheet and entered into one of the earliest relational databases (R-Base) running in DOS. Over the next decade, accommodating a move to the Palo Alto VAHCS and Stanford, data collection evolved into a more convenient human interface using a scan sheet and Microsoft Access™ (Redmond, Washington) running in Windows. The scan sheet was read on a simple scanner and translated using OCR software (PaperKeyBoard by DataCap) (22) and then the data were corrected on graphic user interface (GUI) screens on a personal computer. MUMPS fileman (the computer language used by DHCP) routines were written to provide detailed outcomes of subjects who had undergone an exercise test at both institutions.

The Access program provided the capability of producing a sophisticated report and to add expert system features such as prompts, help, and field definitions (23). Equations were added to report risk estimations using Framingham, Duke, VA, and other prognostic scores (24–26) along with exercise test responses such as percentage of age-predicted exercise capacity using a VA-specific equation developed from the VETS database (27). Angiographic results from cardiac catheterization were computerized and included in the database. Students and visiting faculty helped combine the data sets so that statistical techniques could be applied. Early studies applying VETS data involved assessing the associations between exercise test responses and angiographic findings, including studies on silent ischemia (28), exercise-induced ventricular tachycardia (29), the effect of resting ECG abnormalities on ECG changes during exercise (30), the poor specificity of ST segment changes isolated to the inferior leads (31), the effect of beta blockade on the interpretation of the exercise ECG (32), the prognostic power of exercise capacity (33), nomograms for exercise capacity (27), exercise-induced hypotension (34) and the development of VA scores which became widely applied and recommended in guidelines on exercise testing (26, 35, 36).

**Figure 1:**
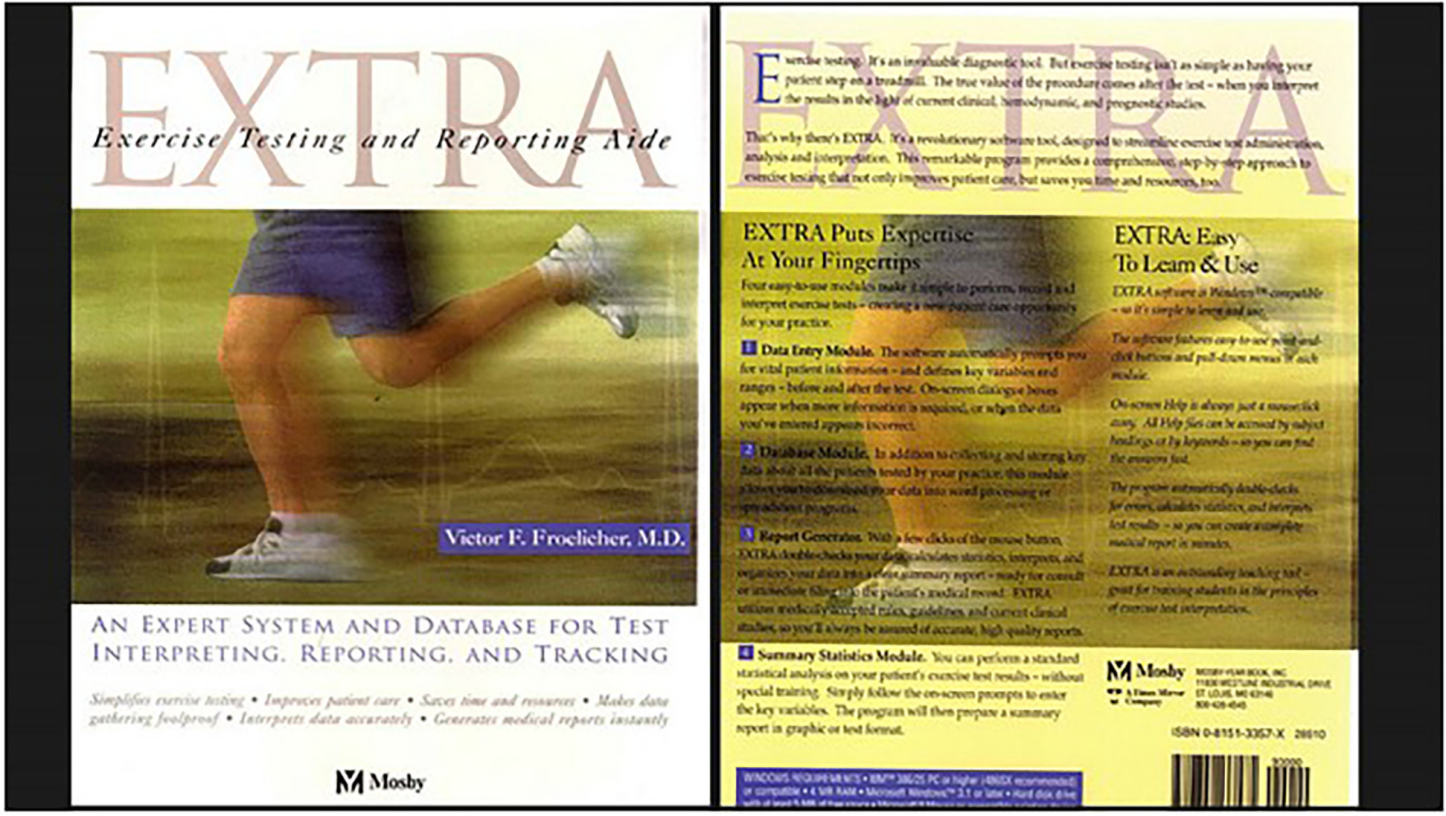
Exercise test reporting aide (EXTRA) released by Mosby publishers in 1996. Reproduced with permission from “Extra: Exercise Testing and Reporting Aide” by Victor Froleicher, 1996.

Later, as the VA electronic medical records were modernized in the 1990s and clinical, demographic, and outcome information were available in the VA Computerized Record System (CPRS), the VETS data set was expanded, and outcomes were continuously updated. The VETS data set was influential in helping to formulate standards and guidelines for exercise testing. A software system based on this approach; termed EXTRA™ (Exercise Test Reporting Aide) was programmed in Visual Basic by Froning and Froelicher (23) to immediately provide test results for physicians conducting the test (Figure 1). These results included the Duke and VA treadmill scores, along with probabilities of CAD. This encouraged the physicians overseeing the tests to enter the data correctly because they would sign the report. A gateway was created into CPRS, so that the text file created by Access was directly downloaded into the treadmill report fields. This approach was employed by multiple other VA systems. Beginning in 2007, we collaborated with researchers at the Washington DC VA, and formed the ETHOS cohort.

### The ETHOS cohort

Data for the ETHOS cohort have been derived from VA exercise labs across the US, leveraging the VA Information Resource Center (VIReC), a resource for developing, disseminating and applying VA data. Detailed information on relevant demographic, clinical and medication information, risk factors, and comorbidities as defined by International Classification of Diseases-9th Revision and International Classification of Diseases-10th Revision coding, with at least 2 recordings at least 6 months apart, are obtained for all participants at the time of the exercise test. Historical information in ETHOS includes the onset of previous MI, cardiac procedures, heart failure, hypertension, type 2 diabetes mellitus (DM2), hypercholesterolemia, cancer (all), renal disease, stroke, smoking status (current and past), and details on cardiac medications. Data extraction and all analyses are performed in accordance with the Strengthening the Reporting of Observational Studies in Epidemiology reporting guidelines for cohort studies (37).

To extract CRF in metabolic equivalents (METs), CPT codes have been applied (93,015–93,018) to identify Veterans who were referred for an exercise treadmill test in VA hospitals across the US beginning in 1999. From this pool, clinical notes associated with the test were identified (*N* > 830,000). From these subjects, 10,000 samples of physician clinical notes on exercise capacity were extracted and peak METs were identified. The notes were preprocessed by snipping each note 30 characters before and after the occurrence of “METs” or “MET level” to extract the relevant context. A simple neural network model was trained on the annotated dataset for 10 epochs, achieving a test accuracy of 98.82%. Spot checks were conducted to verify the accuracy of the model's predictions, and METs were extracted. Duplications, missing values or MET levels <2 or >24 were excluded; the latter MET values were extremely rare and were assumed to have been due to error.

The ETHOS cohort verifies deaths from the VA Beneficiary Identification Records Locator Subsystem. This system, used to determine benefits to survivors of Veterans, has been shown to be 95% complete and accurate. Due to VA regulations, the data set is not currently publicly available.

## Discussion

In the following, some examples in which the VETS and ETHOS cohorts have been applied to assess outcomes related to chronic disease are reviewed.

### CRF and mortality risk

The concept that there is an inverse relationship between higher physical activity patterns and mortality precedes modern epidemiologic methods (38, 39). However, documentation of the relationship between objective measures of CRF derived from a maximal exercise test and mortality is relatively recent; such studies were not available until the 1980s. In a seminal study from the Aerobics Center Longitudinal Study (ACLS), Blair et al. (40) studied CRF by treadmill performance in >13,000 men and women and followed them for >110,500 person-years for all-cause mortality. Mortality rates were lowest among the most fit men (18.6 per 10,000 person-years) and highest (64.0) among the least fit men, with the corresponding rates among women 8.5 and 39.5 per 10,000 person-years, respectively. While these findings closely parallelled results from early studies relating physical activity levels to mortality (38, 39), they were among the first to address the skepticism regarding studies based on highly subjective self-reported physical activity. However, the relatively young, mostly White, and highly affluent participants in the ACLS cohort raised questions regarding the relatively low mortality rates, especially in women, and the universal applicability of the findings.

**Figure 2:**
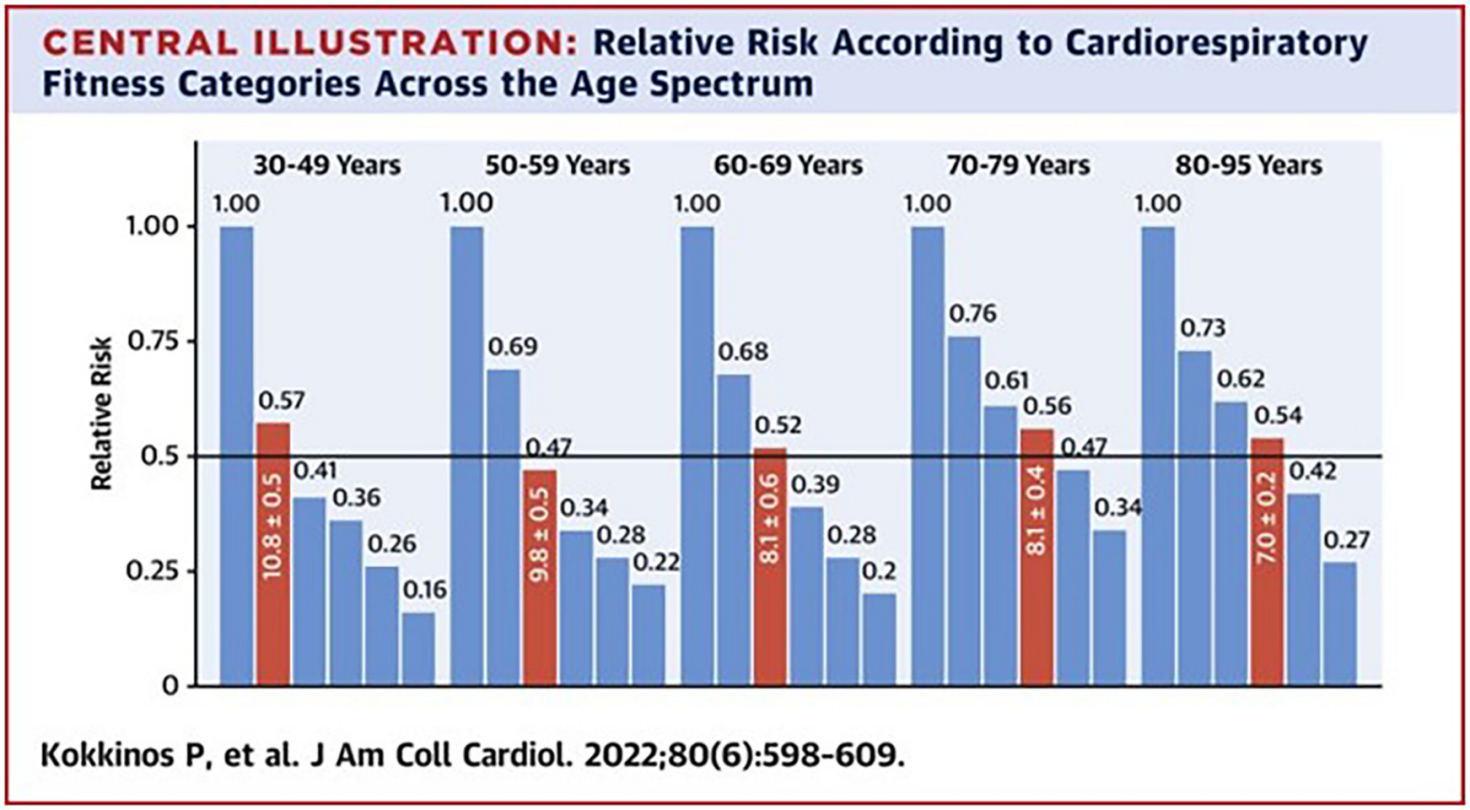
Bars from left to right within each age group represent the cardiorespiratory fitness (CRF) categories on the basis of the percentile of age-specific peak METs achieved (≤20%, 21%–40%, 41%–60%, 61%–80%, 81%–97%, and ≥98%). The mortality risk associated with CRF within each age category is depicted by the HRs (numbers) above each bar. Red bars represent the CRF category for each age group and the peak METs required for approximately 50% lower mortality risk. Reproduced with permission from “Relative Risk According to Cardiorespiratory Fitness Categories Across the Age Spectrum” by Peter Kokkinos, Charles Faselis, Immanuel Babu Henry Samuel, Andreas Pittaras, Michael Doumas, Rayelynn Murphy, Michael S. Heimall, Xuemei Sui, Jiajia Zhang, and Jonathan Myers.

These concerns were addressed by several publications from the VETS and later, the ETHOS cohorts. In an early publication from the VETS cohort (*n* = 6,213 men) (41), an inverse and graded association between CRF and mortality was observed among patients both with and without CVD. This was one of the first studies to demonstrate that mortality risk was progressively elevated with lower CRF levels even in the presence of and after adjustment for other comorbidities and traditional risk factors. More recently, a series of studies from the larger ETHOS cohort extended these findings. In parallel with findings from the VETS cohort, poor CRF was the strongest predictor of mortality when compared to more traditional risk factors, including hypertension, smoking, lipid abnormalities and obesity (42, 43). Second, the adjusted all-cause mortality risk was progressively lower with higher CRF regardless of race, gender, and age, even in octogenarians. The latter observation was unique in that few data had previously been available among individuals >80 years, and the ETHOS cohort included ≈27,000 subjects in their 80s and 90s (44). Third, a peak MET level necessary for a 50% reduction in mortality risk was noted for each decade (30–95 years); simply an average exercise capacity for a given age dropped mortality risk by 50% (Figure 2).

Although the impact of CRF independent of other comorbidities is well-recognized, the inextricable genetic influence on the CRF-mortality risk association presents challenges. Using >93,000 subjects with serial tests in the ETHOS cohort, it was hypothesized that if genetics were the dominant determinant of mortality risk, then changes in CRF over time would have no considerable effect on that risk (45). Mortality risk was examined according to changes in CRF over a mean of 5.8 ± 3.7 years between tests. It was observed that changes in CRF reflected inverse and proportional changes in mortality for both those with and without CVD, regardless of baseline CRF status and independent of other comorbidities. These findings make a persuasive argument that CRF is a strong and independent determinant of all-cause mortality risk, at least partially independent of genetic factors.

### CRF-statin interaction and mortality patients with dyslipidemia

The use of statins to manage dyslipidemia is well-established (46), but many patients cannot tolerate stains because of side effects (mostly muscle pain). Thus, the ETHOS cohort was used to assess the separate and combined effects of statin treatment and CRF on all-cause mortality in 10,043 US Veterans with dyslipidemia (47). Of these, 5,033 were on statin therapy and 5,010 were not. All participants were assigned to one of four CRF categories (Least-fit, Moderately-fit, Fit, and Highly-fit) based on CRF achieved. Over a median follow-up of 10.0 years, mortality risk was lower in those taking statins vs. those not taking statins (18.5% vs. 27.7%; *p* < 0.0001). When mortality risk was assessed across CRF categories, the risk declined progressively with higher CRF among both those treated and not treated with statins.

**Figure 3:**
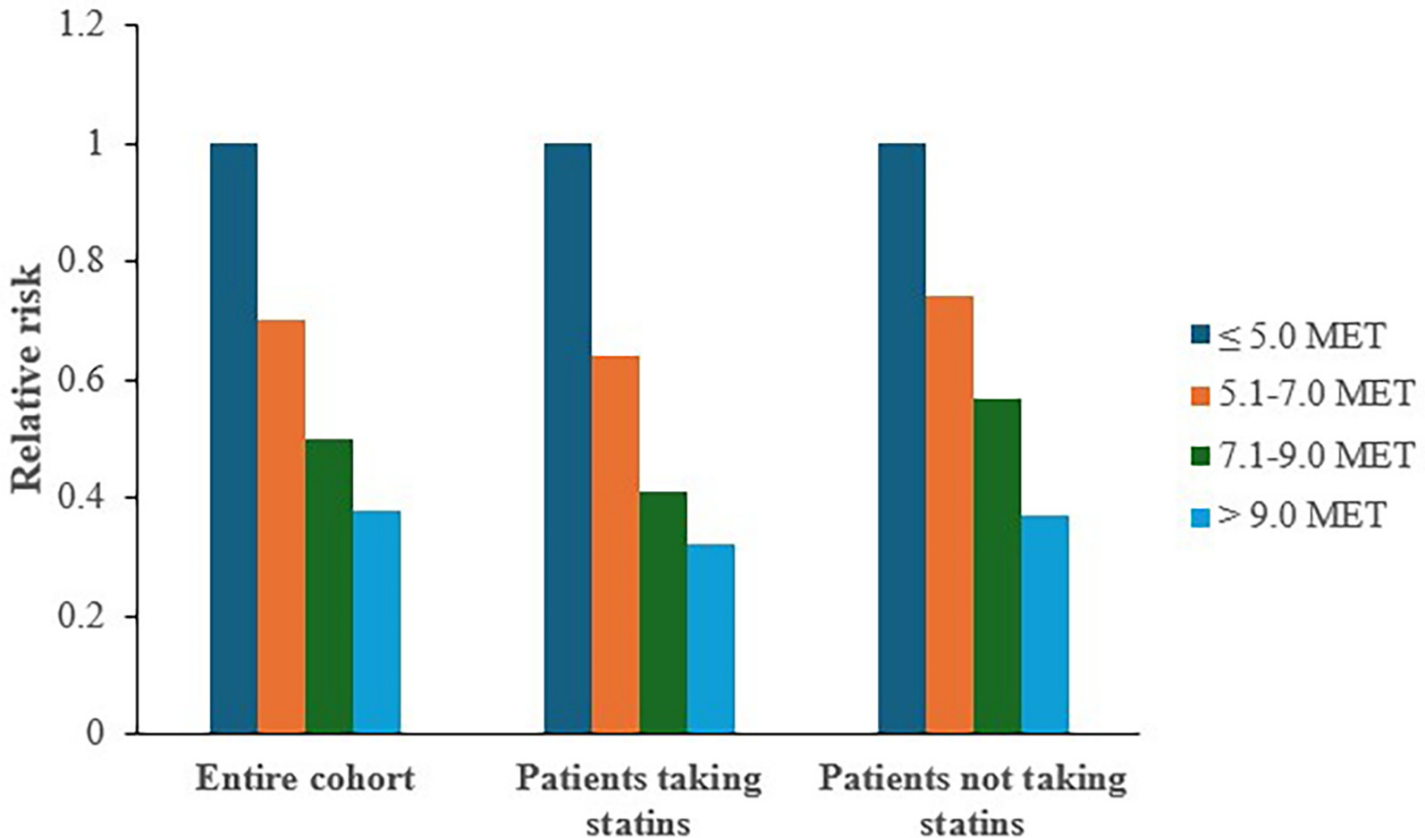
Relative mortality risk by fitness category among patients taking and not taking statins. Adapted with permission from “Relative mortality risk by fitness category among patients taking and not taking statins” by Prof Peter F Kokkinos, Charles Faselis, Prof Jonathan Myers, Demosthenes Panagiotakos and Michael Doumas, licensed under CC BY-NC-ND.

To evaluate the interaction between statin therapy and CRF, mortality risk was assessed for those treated vs. not treated with statins within each CRF category. The Least-fit individuals on statin therapy served as the reference groups in these comparisons. Least-fit individuals not treated with statins had a 35% higher risk of death compared to those treated with statins. The risk then declined progressively with increased CRF; mortality risk was 47% lower for those in the highest CRF category (Figure 3). Collectively, these findings suggest that statin treatment and increased fitness are independently associated with lower mortality among dyslipidemic individuals. The combination of statin treatment and increased fitness resulted in considerably lower risk of mortality than either alone. The higher risk observed for patients in the least fit category not treated with statins suggests that statin therapy is more effective in lowering mortality among those with relatively low fitness.

### CRF and cardiovascular events

In a cohort of >20,500 Veterans, the impact of CRF on the incidence of major adverse cardiac events (MACE) was assessed (48). MACE was defined as the initial occurrence of MI (fatal and nonfatal), heart failure (HF), cerebrovascular accident (fatal and nonfatal), or coronary artery bypass graft surgery. Increased CRF was inversely associated with the risk for MACE independent of other comorbidities. When the risk of MACE was assessed according to an age-specific CRF threshold (6.0 METs; Referent) the risk was 41% higher for those with an exercise capacity 1.0–2.0 METs below the threshold and 95% higher for those with an exercise capacity >2.0 METs below the threshold. Conversely, the risk declined precipitously for those with a CRF level of 6.0 METs or higher. Specifically, the risk was 23% lower for those with an exercise capacity 1.0–2.0 METs above the threshold and 43% lower for individuals with an exercise capacity >2.0 METs above the threshold.

### CRF and heart failure incidence

Among >20,000 male Veterans with no evidence of ischemia or heart failure at a baseline maximal exercise test, the influence of CRF on HF risk and the interaction between HF, body mass index (BMI) and CRF was examined (49). This analysis demonstrated that obese individuals had a 22% higher risk of developing HF compared to normal weight individuals. However, the risk was attenuated when CRF was considered, suggesting that CRF had a modulatory effect on the BMI-HF association. In a subsequent analysis, the impact of CRF on the BMI-HF risk association was examined by quantifying the impact of CRF within BMI categories. There was an inverse and graded association between CRF and risk for developing HF at all levels of BMI independent of comorbidities.

In a subsequent larger cohort of US Veterans (*n* = 624,551) with no evidence of HF or myocardial infarction prior to completion of an exercise test, the risk of developing HF with preserved ejection fraction (HFpEF) was assessed according to CRF (50). A progressive decline in the incidence of HFpEF occurred with higher CRF levels (in a dose-response manner), independent of age, gender, race, CVD risk factors, and medications. Changes in CRF between the initial and final exercise test reflected proportional changes in HFpEF risk. When compared to those who were unfit at both baseline and final evaluations (unfit-unfit), the risk of developing HFpEF was 37% lower for unfit individuals who improved their CRF status to the moderately fit category. Fit individuals who remained fit had the lowest risk. Notably, the protective effect of CRF deteriorated for fit individuals who became unfit, defined as a decline in CRF by approximately 34% over a mean of 6.5 ± 3.9 years between the initial and final ETT evaluations.

### CRF and hypertension

In a cohort of 2,303 pre-hypertensive Veterans followed for ≈9.2 years, we observed that higher CRF was inversely associated with the rate of progression to HTN. Compared to individuals with the highest exercise capacity (>10 METs), the multivariate-adjusted risk for developing HTN was 36% higher for those with an exercise capacity between 8.6 and 10 METs; 66% higher for those between 6.6 and 8.5 METs, and 72% higher for individuals who achieved ≤6.5 METs (51).

### CRF and incidence of type 2 diabetes mellitus

The preventive impact of CRF on the development of Type 2 diabetes mellitus (T2DM) has been described by at least two randomized trials (52, 53). Recently, concerns have been raised regarding the diabetogenicity of statin therapy (54, 55). T2DM patients treated with statins have been shown to progress to insulin therapy at a higher rate than those not treated with statins (56). In the ETHOS study, dyslipidemic patients treated with statins similarly had an increased risk of developing T2DM (57). However, this increase was only evident in patients with relatively poor CRF. We recently noted that the statin-related progression to insulin therapy in T2DM patients treated with statins was accentuated by excess body weight but mitigated by increased CRF regardless of BMI (58). To evaluate the role of CRF on the impact of statins on progression to need for insulin, the ETHOS data set was similarly leveraged to examine this risk within CRF categories among those treated and not treated with statins. For those not on statin therapy, the association between CRF and the rate of progression insulin was inverse and graded. The incidence was 45% lower for those in the highest fit quintile compared to the least fit quintile. For the statin-treated subgroup, the rate of progression to insulin was 43% higher for the Least-fit and only 9% higher for the next least-fit group. These findings suggest that the diabetogenic impact of statins is evident only in patients with relatively low CRF levels (least fit and low fit), and it is attenuated markedly by increased CRF.

### CRF and chronic kidney disease

Chronic kidney disease (CKD) is among the three costliest health conditions in the VAHCS, due to the high costs associated with managing complications, dialysis, and related treatments such as concomitant cardiovascular disease and hospitalizations. Therefore, preventing factors that lead to CKD, such as hypertension, smoking, obesity, and diabetes, can reduce the burden that this condition places on the VAHCS health system. Low CRF is another factor, although largely unrecognized, that strongly influences risk for CKD. Among subjects from the ETHOS cohort who were apparently healthy at baseline, a 22% lower risk for the development of kidney disease was observed for every 1-MET increase in exercise capacity (59). The most fit quartile had a 58% lower risk of developing CKD compared to the least-fit quartile.

### CRF and atrial fibrillation (AF)

Some recent evidence suggests there is a higher prevalence of AF in middle-aged and older elite athletes and those participating in long-term physical activity, particularly high-intensity physical activity, as compared to the general population (60–62). However, this has not been a consistent finding, as an increase, decrease, and no association between low-to moderate-intensity physical activity and the incidence of AF have been reported. While evidence suggests that higher CRF reduces the incidence of AF (63), the evidence is not entirely consistent (64). In the ETHOS cohort, 722 (12.1%) of 5,962 individuals developed AF (14.5 per 1,000 person-years) over a median follow-up of 8.3 years (65). CRF was inversely related to AF incidence. The risk was 21% lower for each 1-MET increase in exercise capacity. Compared with the least fit individuals, hazard ratios were 0.80 for moderately fit individuals, 0.55 for fit individuals, and 0.37 for highly fit individuals.

Individuals who are overweight or obese also have increased risk for developing AF. In a subsequent analysis, obese and severely obese subjects had 13% and 32% higher risks for incidence of AF, respectively, vs. normal weight subjects (66). Overweight and obese subjects in the most fit quartile had a 50% lower risk of developing AF compared to the least-fit subjects. Severely obese subjects had marked increases in AF risk (∼50%–60%) regardless of fitness level. Thus, risk of developing AF increases with higher BMI and lower CRF.

### CRF and health care costs

**Figure 4:**
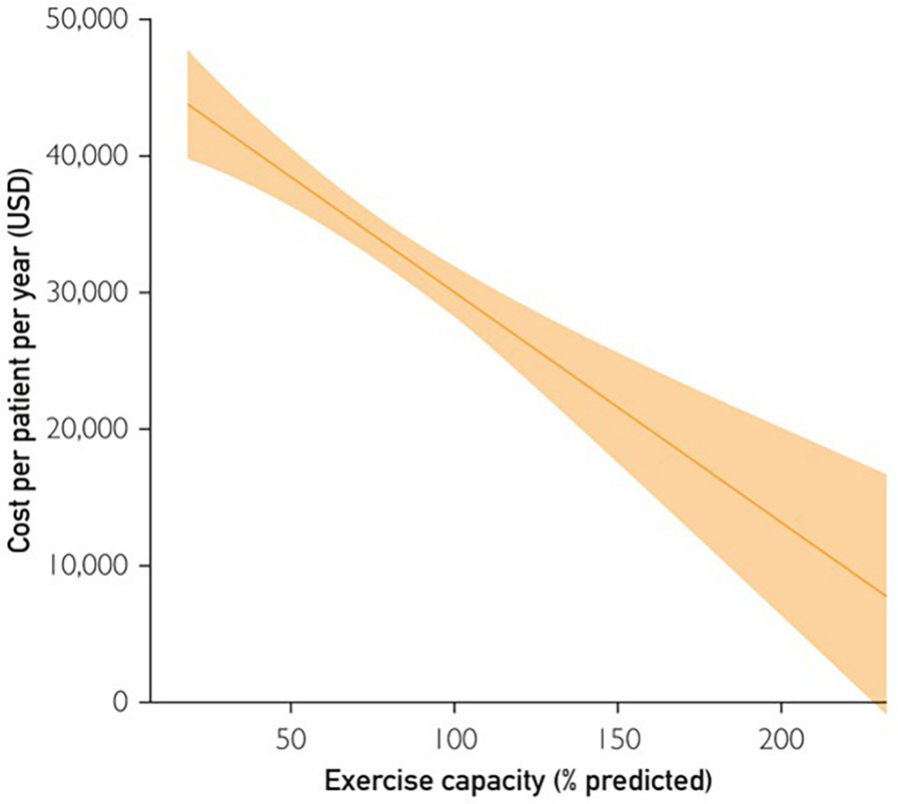
The association between costs per patient per year (USD) and exercise capacity (% age-predicted). USD, US dollars. Reproduced with permission from “The association between costs per patient per year (USD) and exercise capacity (% age-predicted)” by Jonathan Myers, Rachelle Doom, Robert King, Holly Fonda, Khin Chan, Peter Kokkinos and David H. Rehkopf, licensed under CC BY-NC-ND.

There are surprisingly few studies that have been performed on the impact of CRF on healthcare costs. This is likely due to the vicissitudes of healthcare costs, the difficulty obtaining them in a valid fashion, and the fact that CRF is underappreciated as a risk factor (3–5, 67). In the VETS cohort, 9,942 subjects (mean 59 ± 11 years) underwent a maximal exercise test for clinical reasons over a 7-year period (68). CRF, expressed as a percentage of age-predicted peak METs achieved, was categorized in quartiles. Total and annualized healthcare costs were derived from the VA Allocated Resource Center and were compared using multiple regression, controlling for demographic and clinical characteristics. A gradient for reduced healthcare costs was observed as CRF increased (Figure 4), with subjects in the least-fit quartile having approximately $14,662 higher overall costs per patient/year compared to those in the fittest quartile, after controlling for potential confounding variables (*p* < 0.001). Each 1-MET higher increment in fitness was associated with a $1,592 annual reduction in healthcare costs. This equated to a 5.6% lower cost per MET achieved. Most notably, each higher quartile of fitness was associated with a $4,163 annual cost reduction (USD) per patient. The effect of CRF was more pronounced among subjects without cardiovascular disease (CVD), suggesting that the results were not driven by the possibility that less fit individuals had greater CVD. In a model including historical, clinical and exercise test responses, heart failure was the strongest predictor of healthcare costs, followed by CRF (*p* < 0.01).

### Estimating CRF using non-exercise data

The body of evidence from epidemiological studies on the prognostic utility of CRF has led the AHA to recognize CRF as a vital sign that should be considered during clinical encounters, much like the routine assessment of blood pressure (3). However, routinely conducting a maximal exercise test on all asymptomatic individuals is neither feasible nor recommended (69). This has presented a quandary regarding how to incorporate CRF into routine clinical practice. A growing number of efforts have addressed this issue by applying multivariate estimates CRF using non-exercise data. These studies have included factors such as age, BMI, gender, risk factors, physical activity patterns and others. Multiple R values associating estimated CRF with measured CRF have been reported in the range of 0.80–0.90, and these models have been widely applied in epidemiologic studies (70). Additional details on this topic are beyond the scope of the current review, but have recently been reviewed elsewhere (3, 4, 70).

## Summary

For more than a quarter century, the VA cohorts described herein have been influential in helping to define the role of CRF in determining health outcomes, including prevention of chronic disease, incidence of cardiovascular events, and all-cause mortality. Higher CRF has a favorable impact on outcomes across the spectrum of chronic conditions. These studies, along with multiple contemporaneous trials, have helped formulate and update guidelines on exercise testing as well as recommendations from national and international health organizations on physical activity. These observational studies underscore the need for greater efforts to reduce sedentary behavior, promote physical activity, and enhance CRF by public health organizations and healthcare systems in order to reduce the risk of chronic disease.

## Data Availability

Requests to access the datasets can be directed to pk543@kines.rutgers.edu.
